# Radiation Damage in Biomolecules and Cells 3.0

**DOI:** 10.3390/ijms25126368

**Published:** 2024-06-08

**Authors:** Mario P. Carante, Ricardo L. Ramos, Francesca Ballarini

**Affiliations:** 1 Physics Department, University of Pavia, Via Bassi 6, I-27100 Pavia, Italy; mariopietro.carante@unipv.it; 2 INFN (Italian National Institute for Nuclear Physics), Sezione di Pavia, Via Bassi 6, I-27100 Pavia, Italy; ricardoramos85@gmail.com

Ionizing radiation is widely used in medicine, not only as a diagnostic tool but also as a therapeutic agent, since about half of cancer patients are treated with ionizing radiation, while most of them are irradiated with X-rays. Cancer ion therapy is now more prevalent (e.g., [1,2]). At the same time, several exposure scenarios, including environmental and occupational exposure, as well as astronauts’ exposure to space radiation (e.g., [3,4,5]), raise radiation protection issues. The scientific community is therefore continuously improving its knowledge of the various biophysical mechanisms underlying the induction of radiation effects in biological molecules and cells, and the acquired information can be applied to optimize both the use of ionizing radiation in medicine and the strategies that can be adopted in radiation protection. DNA is widely recognized as the main radiation target, although phenomena such as the bystander effect [6] play a non-negligible role at very low doses. Incorrect repair of the initial DNA damage can lead to various types of chromosome aberrations (e.g., [7,8]), some of which may lead to cell death, while others may cause the cell to become malignant [9]. All these processes depend not only on radiation dose but also on several other factors, including dose rate, radiation type and energy, target-cell radiosensitivity, cell cycle phase, etc.

In this framework, this Special Issue reports experimental and theoretical works on the effects of ionizing radiation at the DNA level, as well as possible applications in cancer therapy and space radiation protection. More specifically, Kundrat et al. [10] applied the PARTRAC simulation code to perform track-structure-based simulations analyzing DNA damage induction by different isotopes occurring in nuclear reactions involved in ion-beam radiotherapy, cosmic-ray shielding, and dating techniques. A database of simulations was presented for H, He, Li, Be, B, and C isotopes at energies from 0.5 GeV/u down to stopping; the doses deposited in a cell nucleus, as well as the yields of DNA single- and double-strand breaks and their clusters, were predicted to vary among diverse isotopes of the same element at energies < 1 MeV/u, especially for isotopes of H and He. The results may affect the risk estimates for astronauts involved in deep space missions and/or the models of ion-beam biological effectiveness and indicate that radiation protection in ^14^C or ^10^Be dating techniques may be based on knowledge gathered with ^12^C or ^9^Be.

Moreover, by means of a theoretical approach, Karwowski [11] analyzed the influence of spirodi(iminohydantoin) on charge transfer through ds-DNA containing 8-OXO-dG, a DNA damage event that can undergo further transformations towards spirodi(iminohydantoin) (Sp), which can be highly mutagenic in comparison to its precursor if not repaired. The results indicated that DNA damage such as spirodi(iminohydantoin), especially when becoming part of clustered DNA damage, can affect the effectiveness of other lesion recognition and repair processes, which in turn can lead to the acceleration of processes such as carcinogenesis or aging. At the same time, the slowing down of the repair machinery can result in increased effectiveness in terms of oncological radiotherapy, chemotherapy, or combined therapy. More generally, the influence of DNA clustered damage on charge transfer, as well as its subsequent effects on single-damage recognition by glycosylases, require future investigation. At the cellular level, Guerra and McMahon [12] characterized the intrinsic radiosensitivity in a wide panel of normal, cancerous, and CRISPR-modified cell lines. The cell characterization was performed by measuring a range of biological features, including the induction and repair of DNA double-strand breaks (DSBs), cell cycle distribution, ploidy, and clonogenic survival following X-ray irradiation. These results were used to investigate correlations between potential radiosensitivity determinants, finding a wide variation in the intrinsic radiosensitivity of cell lines. While the data provided a valuable dataset for the modeling of radiobiological responses, the differences in the predictive power of residual DSBs between CRISPR-modified and other subgroups suggest that genetic alterations in other pathways, such as proliferation and metabolism, may have a greater impact on cellular radiation response. 

In the framework of cancer radiotherapy research, Nowak et al. [13] investigated how to improve the radiosensitivity of lung cancer cells based on Chinese medicine and/or conventional medicine pharmacy drugs by reviewing potential candidates that may show a radio-sensitizing effect on lung cancer cells. Finally, concerning radiation-induced damage at the tissue, organ, and organism level, Ramos et al. [14] predicted astronauts’ doses in the event of exposure to cosmic rays during a long-term mission in deep space, such as a future journey to Mars. The authors exploited an interface between the FLUKA Monte Carlo radiation transport code and the BIANCA biophysical model, which allowed calculating both the RBE (Relative Biological Effectiveness) for cell survival, which is related to non-cancer effects, and that for chromosome aberrations, related to the induction of stochastic effects including cancer. Comparisons with the astronauts’ dose limits suggested that a 650-day Mars mission at solar minimum would respect the 1 Sv career limit recommended by the International Commission on Radiological Protection (ICRP), but would not respect the 600 mSv limit recently adopted by NASA.
